# Bimaxillary simultaneous immediate loading of full-arch restorations: A case series

**DOI:** 10.4317/jced.54172

**Published:** 2017-09-01

**Authors:** Iñaki Cercadillo-Ibarguren, Alba Sánchez-Torres, Rui Figueiredo, Eduard Valmaseda-Castellón

**Affiliations:** 1 DDS, MS, Master of Oral Surgery and Implantology. Professor of the Master of Oral Surgery and Implantology degree program, School of Medicine and Health Sciences, University of Barcelona. Researcher at the IDIBELL institute; 2 DDS. Fellow of the Master of Oral Surgery and Implantology degree program, School of Medicine and Health Sciences, University of Barcelona; 3 DDS, MS, PhD, Master of Oral Surgery and Implantology. Associate professor of Oral Surgery and Professor of the Master of Oral Surgery and Implantology degree program, School of Medicine and Health Sciences, University of Barcelona. Researcher at the IDIBELL institute; 4 DDS, MS, PhD. Master of Oral Surgery and Implantology. Professor of Oral Surgery. Director of the Master of Oral Surgery and Implantology degree program, School of Medicine and Health Sciences, University of Barcelona. Researcher at the IDIBELL institute

## Abstract

**Aim:**

To describe a bimaxillary simultaneous immediate loading protocol with full-arch implant-supported fixed prostheses.

**Material and Methods:**

A prospective case series of 8 patients who required full-arch rehabilitation was conducted. The main inclusion criteria were patients with teeth that required extraction. At least 1 molar per arch was temporarily employed to stabilize the surgical template and the provisional prosthesis during intraoral relining.

**Results:**

Two upper implants failed in 1 patient. Structural fracture was registered in 3 patients, around 3 months after loading. All of them had bruxism. Three esthetic complications were registered: midline deviation, canting of the oclusal plane and color mismatch.

**Conclusions:**

Although this protocol achieves optimal results, some mechanical complications were encountered. The fracture of the provisional prosthesis is a relatively common mechanical complication but does not seem to jeopardize the final treatment result.

** Key words:**Implant-supported full-arch, provisional prosthesis fracture, bimaxillary simultaneous rehabilitation, conical abutments.

## Introduction

Implant-supported rehabilitations are a predictable therapeutic option (1-5). Treatment protocols have evolved since the introduction of dental implants around the 1970s, and today it is common to place and load the implants immediately, allowing the patient to regain function a few hours after the surgical procedure (5).

Immediate loading of full-arch fixed prostheses has survival and complication rates similar to early or conventional loading (6). An insertion torque of 30 N•cm or higher is mandatory when performing an immediate loading protocol (6,7). The scientific literature shows no difference on success rate between immediate postextraction or conventional placing of implants under immediate loading protocols (4,8,9)

However, the presence of risk factors can affect the implant survival rates and the occurrence of complications (1,10-12). Therefore, a multidisciplinary approach is of paramount importance to achieve optimal esthetic and functional outcomes, especially in cases where an esthetic component is involved (13,14).

The aim of this paper is to describe a bimaxillary simultaneous immediate loading protocol with full-arch fixed prostheses.

## Material and Methods

A prospective case series was conducted in a private practice environment. The inclusion criteria were patients aged ≥18 years old, partially edentulous in both arches, with hopeless teeth, at least 1 remaining molar per arch, and a full-mouth plaque and bleeding score of <30%. All the patients were willing to participate in the study and signed an informed consent document. The study protocol was authorized by the ethical review board of the Dental Hospital of the University of Barcelona. The exclusion criteria were general contraindications for oral surgery (such as a history of bisphosphonate therapy, current chemotherapy or radiotherapy of the head and neck), and active infection or acute inflammation in the areas where the implant was to be placed. Advanced periodontal disease was not an exclusion criterion and all the patients underwent active periodontal treatment before implant surgery.

One researcher registered the following data: demographic variables (age, gender, smoking habit, alcohol consumption, history of periodontitis, bruxism or use of an occlusal appliance), intraoperative data (number of implants placed in each arch and the need for guided bone regeneration) and postoperative variables (mechanical, biological and esthetic-functional complications, and time wearing the provisional prosthesis).

**Figure 1:**
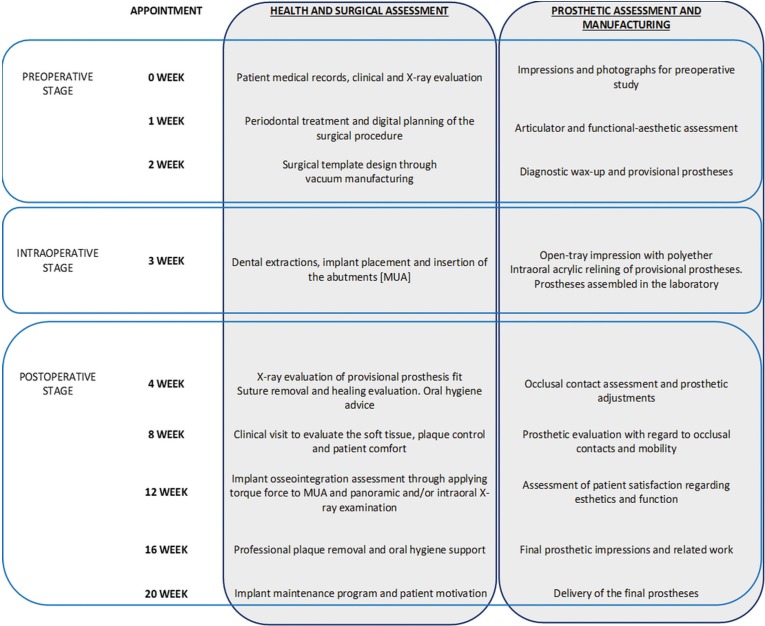
Protocol. Description of surgical and prosthetic protocol.

Figure 1 describes the main steps in the surgical-prosthetic protocol.

-Esthetic and functional diagnosis

An initial esthetic and functional diagnosis was made. Impressions for the study casts were taken and the intermaxillary relation was recorded using an articulator and facebow (Artex®, Amann Girrbach AG, Austria). Dental proportions were established by means of Chu’s esthetic gauges (Hu-Friedy®, Chicago, USA). Intraoral and extraoral photographs were taken to assess the est-hetic parameters, as well as for color selection.

-Surgical planning

Virtual placement of the implants was performed using NobelClinician™ version 2.4 software (Nobel Biocare AB, Göteborg, Sweden), after initial evaluation of a panoramic radiograph and a computed tomograph (CT).

-Diagnostic wax-up

**Figure 2:**
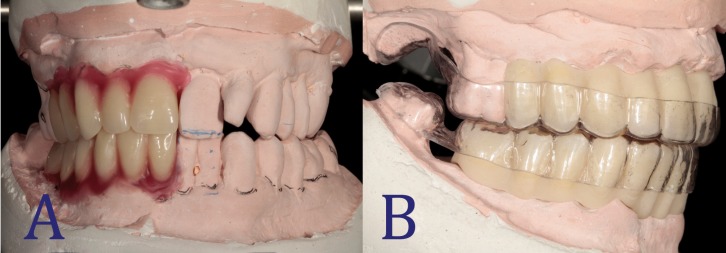
A: Diagnostic wax-up. Diagnostic wax-up oriented by clinical parameters related to esthetics and function. B: Surgical template. Surgical template prepared by vacuum forming over the provisional prosthesis. At least one molar was left in both arches.

**Figure 3:**
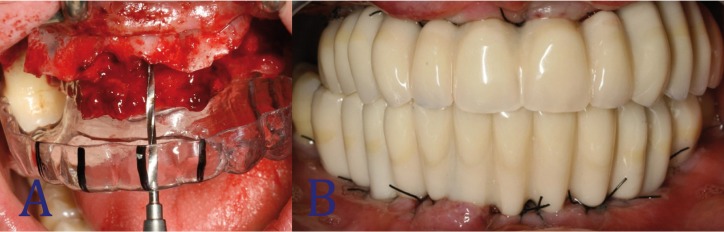
A: Relationship between prostheses and bone architecture. Intraoral view of the surgery placing the surgical template with the provisional prosthesis inside to determine the dimensions of the bony defect and the drilling. B: Provisional prostheses. Final provisional prostheses with ovoid pontics and correct emergence profile.

A diagnostic wax-up was prepared by the dental technician under the supervision of the clinician (Fig. 2A). The provisional prosthesis was then manufactured, based on the wax-up. Through vacuum forming a 1.5 mm acrylic sheet placed over the provisional prosthesis, a surgical template for correct 3D implant placement was obtained (Fig. 2B). At least one molar was left in the arch in order to fix the template in the right position during the surgical procedure (Fig. 3A).

-Surgical procedure

After extracting the remaining teeth, a minimum of 4 implants were placed in each arch (Replace® Tapered, Nobel Biocare AB, Göteborg, Sweden or Inno External CWM®, Cowellmedic Co., Seul, South Corea), aiming to achieve a minimum insertion torque of 35 N•cm. Guided bone regeneration was performed when needed. Postextraction defects and horizontal defects around implants or in pontic areas were treated with autologous bone chips collected from the tuberosity or retromolar area and/or xenografts (BioOss®, Geistlich Biomaterials, Wolhusen, Switzerland) and resorbable bovine collagen membranes (BioGide®, Geistlich Biomaterials, Wolhusen, Switzerland). In cases with esthetic defects in the soft tissues, a connective tissue graft was harvested to offset them. Conical Multi-Unit abutments (MUA®, Nobel Biocare AB, Göteborg, Sweden) were then attached to all the implants. Open-tray impression copings were put in place and the wound was sutured with a 4/0 suture (Supramid®, SMI AG, St. Vith, Belgium) and/or 6/0 suture (Resotex®, Resorba Medical GmbH, Nürnberg, Germany). A polyether impression material was employed (Impregum™, 3M ESPE, Seefeld, Germany).

-Prosthetic treatment

Two provisional titanium abutments were connected to the Multi-Unit abutments in each arch. The provisional prostheses were perforated to match the position of the provisional titanium abutments, placed in their correct position according to the facial, occlusal and intermaxillary references, and relined with self-curing temporary BIS-acrylic material (Structure 2 SC®, VOCO Gmbh, Cuxhaven, Germany). At least one molar per arch was needed to achieve correct seating of the provisional prostheses. After 4-5 minutes, the prostheses were removed and attached to the cast models in order to determine the correct relationship between the two arches. The dental technician then filled the empty spaces with acrylic resin, relined the prosthesis with ovoid pontics and polished all the surfaces (Polishing ARG®; Bredent, Munich, Germany). Screw-retained full-arch acrylic prostheses with standard commercial cast teeth, reinforced with a 1.2 mm wide metal ligature, were placed in position 8 to 24 hours after the surgical procedure (Fig. 3B).

The interproximal spaces were opened slightly to ensure good dental hygiene, and the pontics were ovoid. Finally, the occlusion was adjusted with a canine guidance or group function, with very soft contact of the anterior teeth and the overall esthetic outcome was evaluated.

-Follow-up visits

All the patients were assessed for suture removal 7 to 10 days after the surgical procedure. A panoramic radiograph was taken to check the correct seating of the prostheses. The patients were recalled for clinical evaluation at 45 and 90 days after surgery, unless some unexpected problem occurred. Information regarding potential complications such as prosthesis mobility/fracture were provided and the patients were instructed to call the center immediately to schedule an appointment in such cases. Occlusal stability and balanced contact points were checked at every visit.

-Statistical analysis 

Statistical analysis was performed with SPSS 22.0 software (IBM Corp, Armonk, New York). Descriptive and bivariate analysis (t-Student and Chi-square tests) were performed and *p* values of 0.05 or less were considered statistically significant.

## Results

This case series comprises a total of 8 patients rehabilitated with bimaxillary acrylic full-arch fixed prostheses, using a simultaneous approach. The mean age of the participants was 55.8 years (range 39-73 years) and all the subjects had a history of periodontal disease. A total of 81 implants (71 Replace Select and 10 CWM) were placed in 16 arches. The mean time wearing the provisional prostheses was 5 months (range: 3.5 to 7 months). The main patient data and complications can be seen in  Table 1.

**Table 1:**
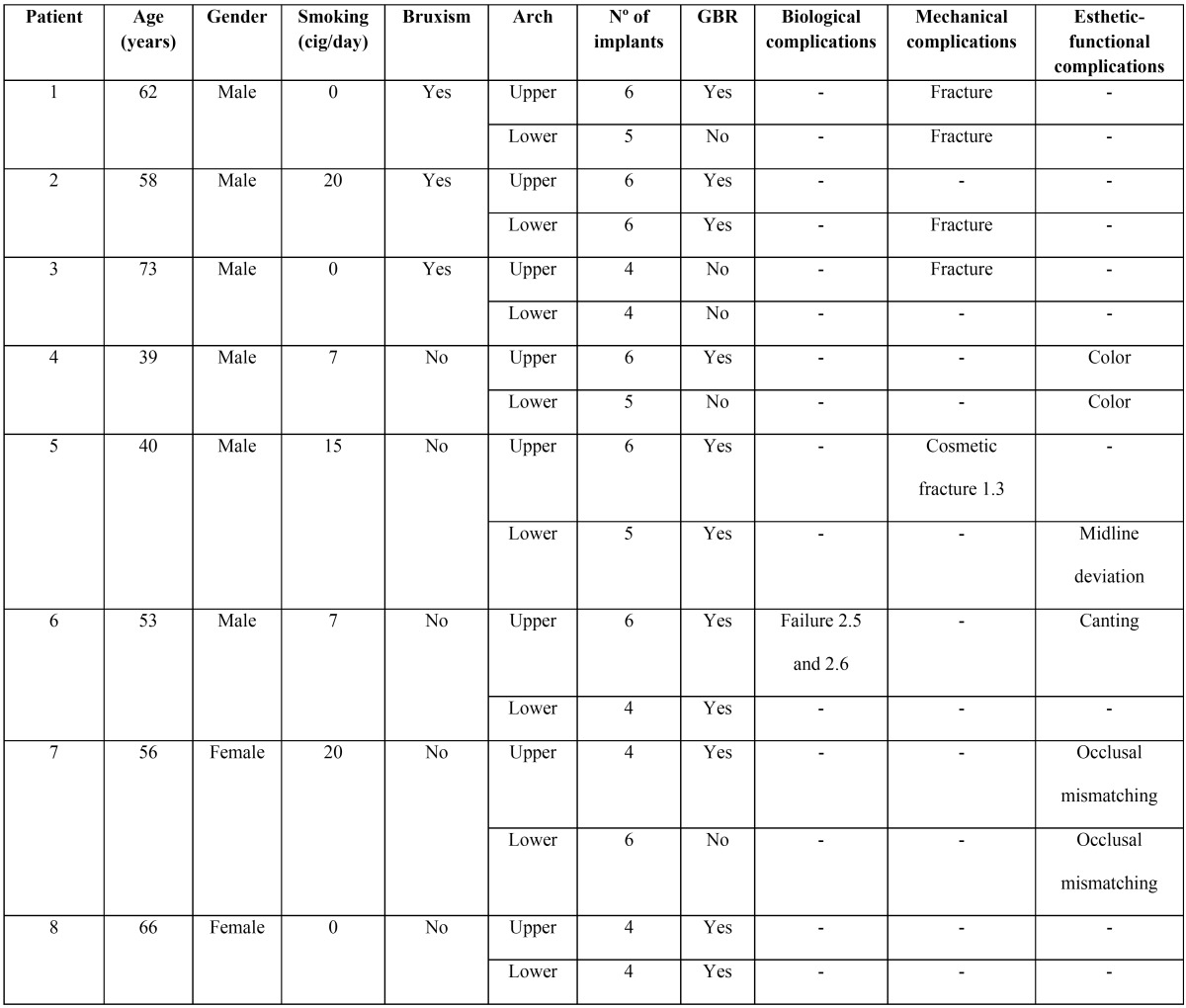
Characteristics of the participants and biological, mechanical and esthetic complications that occurred during the evaluation period of the study.

The failure of 2 upper implants in patient #6 was the only complication that delayed the treatment. These implants were replaced in a single surgical procedure and the provisional prosthesis was then adapted to the position of the new implants.

All the mechanical complications were solved at the dental clinic without needing to send the prostheses to the dental technician. Structural fracture was registered in 3 patients (37.5%), around 3 months after loading in all 3 cases. It is noteworthy that this complication occurred in patients with bruxism, even though all of them wore an occlusal splint.

Three esthetic complications were registered: midline deviation, canting of the oclusal plane and color mismatch. These issues were solved with the definitive prostheses. The occlusal mismatch in one patient was corrected by adding composite material at the occlusal surface to achieve symmetrical, balanced contact.

## Discussion

Immediate loading has some advantages, such as immediate function and esthetics (1,6,8,15), fewer surgical procedures and postoperative visits (1,8,15), reduced treatment time (1,6,16), less psychological, social and work impact (6,17) and improved soft tissue healing (17). However, some complications, like fracture of the provisional prosthesis, can affect the treatment results (3,7,8,17-19). Also, other factors like immediate insertion of the final prosthesis and the wide variety of protocols may alter the therapeutic outcome (17).

The use of a tooth-supported surgical guide is, in our opinion, a key factor for success, since this template is much more stable and facilitates implant placement, especially when compared to guides supported by the mucosa or bone (20,21).

The results of this study and those of several other authors have shown that biological complications are rare events in the short-term (2,3,18). A retrospective study by Butura and Galindo (22), which reported on simultaneous rehabilitation of both arches with immediate loading, yielded a 100% implant survival rate after a follow-up period of 2 years.

Fracture of the provisional prosthesis is the most common mechanical complication, with an estimated incidence ranging from 10% to 11% in single arch restorations (2,3,7,18). In the present sample, approximately one third of patients presented this com-plication. The fact that this study only included patients with full-mouth rehabilitation (both arches) could explain this high figure. Several authors have identified bruxism (2,7), a progressive change from a soft to a regular diet, and wear on the immediate-loading provisional prosthesis as risk factors for this complication (7). Indeed, all the fractures in the present sample occurred in men with parafunctional habits at around 3 months after loading. Wear on the acrylic material and patient comfort (which could predispose to eating a harder diet) could both be among the factors related to prosthetic fractures. Surprisingly, this mechanical complication was not related to osseointegration failures. The 2 implant failures registered were probably related to poor primary stability. Also, these complications still allowed the subjects to wear the immediate-loading provisional prostheses during the entire treatment period.

Digital tools for smile design could improve the final esthetic outcome, since they make diagnosis and communication with both patients and dental technicians simpler (23). Nevertheless, few esthetic complications were found with the present protocol and they were managed easily. In fact, provisional prostheses provide patients and dentists with extremely useful information, making it possible to take any potential flaws identified during the provisional phase into account when constructing the final restoration.

One of the major advantages of immediate loading is that patients report a high level of satisfaction (9,24,25), and even an improvement in their quality of life, compared to the use of removable dentures (25).

The decision to treat one arch or two arches simultaneously depends on several factors. One of the advantages of a bimaxillary approach is that the patient will be treated in a single session. The main disadvantage could be related to the fact that it is a highly demanding procedure that involves a coordinated multidisciplinary approach (oral surgeon, prosthodontist and dental technician). Another possible drawback is the need to perform intravenous conscious sedation or general anesthesia, due to the lengthy operation time. In our opinion, intravenous sedation seems more appropriate since the patient feels more comfortable during and after the surgical procedure and is able to collaborate with the surgeon and prosthodontist during the treatment.

The provisional prostheses are intended to be functional for no more than 6 months and the patient should be aware of the increased risk of mechanical complications if the treatment time is extended. In order to reduce the structural fractures of provisional prostheses, new designs and materials have been suggested, such as milled monolithic structures. Additionally, patient compliance is a key factor and dentists must insist that patients keep to a soft diet and use an occlusal appliance during this provisional phase.

## Conclusions

- Within the limits of the study, this simultaneous bimaxillary immediate loading protocol achieves optimal outcomes in terms of function and esthetics.

- Fracture of the provisional prosthesis is a relatively common mechanical complication but does not seem to jeopardize the final treatment result.
